# Fabrication of PCD Skiving Cutter by UV Nanosecond Laser

**DOI:** 10.3390/ma14144027

**Published:** 2021-07-19

**Authors:** Jianlei Cui, Xuyang Fang, Xiangyang Dong, Xuesong Mei, Kaida Xu, Zhengjie Fan, Zheng Sun, Wenjun Wang

**Affiliations:** 1 State Key Laboratory for Manufacturing Systems Engineering, Xi’an Jiaotong University, Xi’an 710049, China; fxy1997@stu.xjtu.edu.cn (X.F.); lovelyhat@stu.xjtu.edu.cn (X.D.); fanzhengjie@xjtu.edu.cn (Z.F.); zheng.sun@xjtu.edu.cn (Z.S.); wenjunwang@mail.xjtu.edu.cn (W.W.); 2 Department of Project, Xi’an Jiaotong University Shenzhen Research Institute, Shenzhen 518057, China; 3 School of Information and Communications Engineering, Xi’an Jiaotong University, Xi’an 710049, China; kaidaxu@xjtu.edu.cn

**Keywords:** PCD skiving cutter, UV nanosecond laser, contour accuracy, surface morphology

## Abstract

Polycrystalline diamond (PCD) skiving cutter has dominated research in recent years. However, the traditional methods of fabrication have failed to cut the diamond with high quality. We propose the two-step laser machining process combining roughing machining with orthogonal irradiation and finishing machining with tangential irradiation. In addition, the processing effect and mechanism of different lasers on the diamond were investigated by a finite element analysis. It’s proved that the ultraviolet nanosecond laser is an excellent machining method for the processing of diamond. Furthermore, the effect of the processing parameters on the contour accuracy (*R*_t_) was studied. The result indicates that the *R*_t_ value decreases first and then increases as the increase of the line interval, scanning speed and defocusing amount (no matter positive or negative defocus). Further, Raman spectroscopy was applied to characterize the diamond surface under different cutting methods and the flank face of the tool after processing. Finally, a high-quality PCD skiving cutter was obtained with an *R*_t_ of 5.6 µm and no phase transition damage.

## 1. Introduction

The demand of shape accuracy, surface quality, working stability and reliability is increasing incessantly in miniature parts with the development of aerospace, electronics, ultra-precision machinery and other fields [1,2,3,4,5]. The gear is a significant mechanical component that transmits power and movement. The main manufacturing methods for gear include gear hobbing, milling, shaper, broaching, skiving, etc. The skiving method is receiving more and more attention due to its higher efficiency and accuracy. The fabrication of a skiving cutter is the key to obtain a high precision gear.

Polycrystalline diamond (PCD) has the advantages of extremely high hardness (only inferior to single-crystal diamond), high strength, wear resistance and can be sharpened to an extremely sharp blade. The PCD has become one of the most suitable materials for diamond cutting tools. Due to its ultra-high hardness, the traditional cutting methods are unideal to process diamond. However, laser has obvious advantages in processing the diamond due to its excellent properties such as high peak power, machining accuracy and efficiency, non-contact processing, wide processing range [6,7,8,9].

Discussions with respect to the laser processing of the diamond have dominated research in recent years. Butler-Smith et al. [10] reported there is a 2 µm thick graphite layer on the diamond surface after processing with a 10 ns laser, and it has an abrupt boundary with the diamond structure. Harrison et al. [11] used a 200 ns and 1064 nm laser to cut polycrystalline diamond composites, and studied the ablation characteristics, the removal rate of nanosecond laser processing PCD. Everson et al. [12] studied the effect of the repetition frequency and feed rate on PCD slit and surface morphology with a nanosecond laser. According to Konrad et al. [13], a cutting edge with a corner radius of 3 µm and a tolerance within 2 µm on a polycrystalline diamond was obtained used a 10 ps laser. Dold et al. [14] compared the diamond cutting tools processed by picosecond laser with the diamond cutting tools processed by traditional methods. The results indicate that the surface of cutting tools processed by laser processing is smoother. This paper aims to study the fabrication process of PCD skiving cutter by UV nanosecond laser, analyze the difference between different lasers by finite element simulation and Raman detection was carried out to analyze the phase transition of the cutting surface.

## 2. Materials and Methods

**Figure 1:**
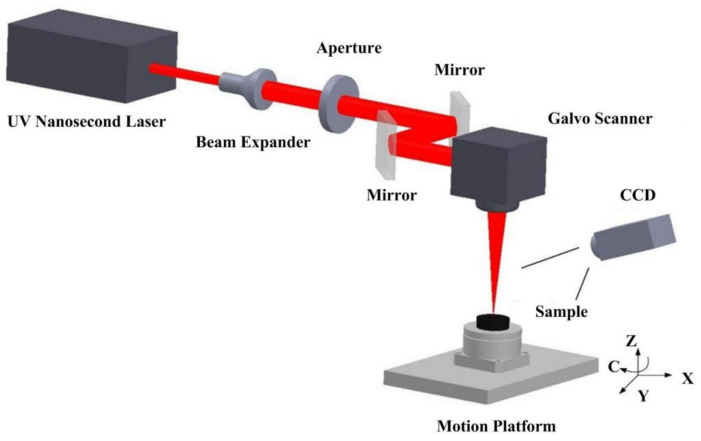
Schematic diagram of laser precision machining system.

A PCD diamond blank with a diameter of 20 mm and a thickness of 2 mm was used as the skiving cutter material sample. In the experiment, the parameters of the incident UV nanosecond laser (INNO-FOTIA-355-OEM-2, Shenzhen, Guangdong, China) are listed in Table 1. Figure 1 shows the schematic diagram of the laser precision machining system. The beam expander can adjust the divergence angle of the laser to obtain a smaller spot on sample surface. Then using an aperture to improve the spot shape of the laser beam. Finally, the laser beam enters into the galvo scanner, which can control the laser light to move along the predetermined scanning path. The sample was fixed on a 4-axis Computer Numerical Control (CNC) platform (XYZ linear and C axis rotary). Furthermore, a Charge Coupled Device (CCD) camera was used to observe the machining process in real-time and adjust the tool.

To ensure the cutting efficiency, the experiment fixed the pulse repetition rate at 50 kHz, at which this laser average power is maximum. We applied a two-step machining method combining roughing machining and finishing machining to realize the high precision of the skiving cutter.

**Figure 2:**
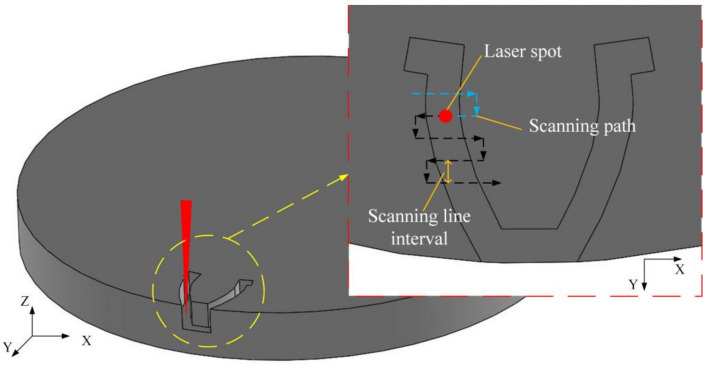
Schematic of laser roughing machining PCD skiving cutter.

**Figure 3:**
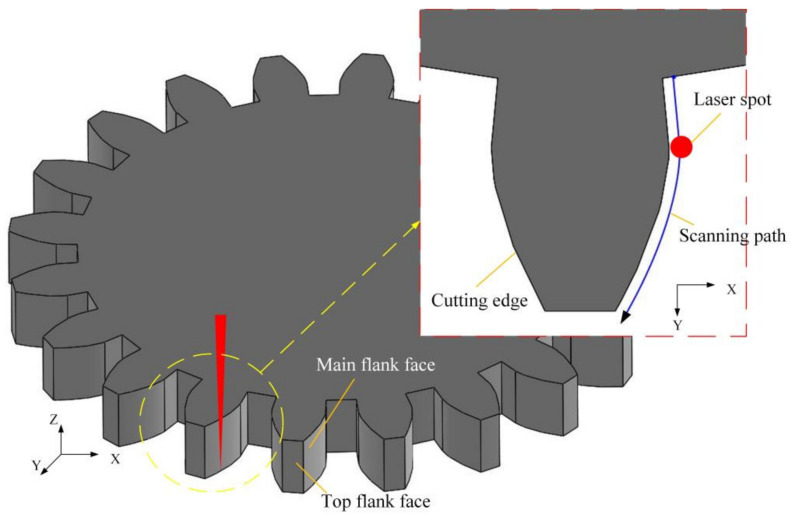
Schematic of laser finishing machining PCD skiving cutter after roughing machining.

During laser roughing machining process, the laser beam transmission direction was vertical to the horizontal plane. During laser finishing machining process, the laser beam was still vertical to the horizontal plane. Between the roughing machining process and the finishing machining process, the PCD workpiece was not rotated or moved, the laser beam was not rotated as well. Therefore, the relative orientation of the reference system determined by the laser beam transmission direction and the reference system determined by the static PCD workpiece was not changed between the roughing machining process and finishing machining process. As is shown in Figure 2 and Figure 3, in laser roughing machining process, laser beam energy was mainly used to etch the PCD material under the focus point of the laser beam. While in the laser finishing machining process, the axial trajectory of the laser beam is basically consistent with the contour the machined outer edge of the gear. In addition, the small outer part of laser beam was mainly used to etch the PCD material. In order to picture the process of laser beam etching the PCD material, the process of laser etching PCD underneath the laser spot during laser roughing and finishing machining process is named orthogonal exposure and tangential exposure in Figure 2 and Figure 3, respectively.

The roughing machining adopted orthogonally irradiation that the direction of the laser beam is perpendicular to the processed surface. Orthogonally irradiation can remove a large amount of material quickly. Meanwhile, the laser beam scanned along the tool contour in an S shape in the horizontal direction (xy plane) to ensure the basic contour shape of the cutting tool, as illustrated in Figure 2. Moreover, an additional experiment was applied to study the effect of processing parameters on the cutting contour accuracy in the roughing machining. It aims to find a superior parameter combination to minimize the minimum grinding allowance of finishing machining. The finishing machining performed tangentially irradiation that the laser beam is parallel to the processed surface. Moreover, the laser beam scanned rapidly with a tool contour path along the cutting edge, as described in Figure 3. Tangentially machining can polish the flank face and further repair the shape of cutting edge. Therefore, it is feasible to create an excellent PCD skiving cutter.

## 3. Results

### 3.1. Simulation Analysis of Diamond under Different Lasers

**Figure 4:**
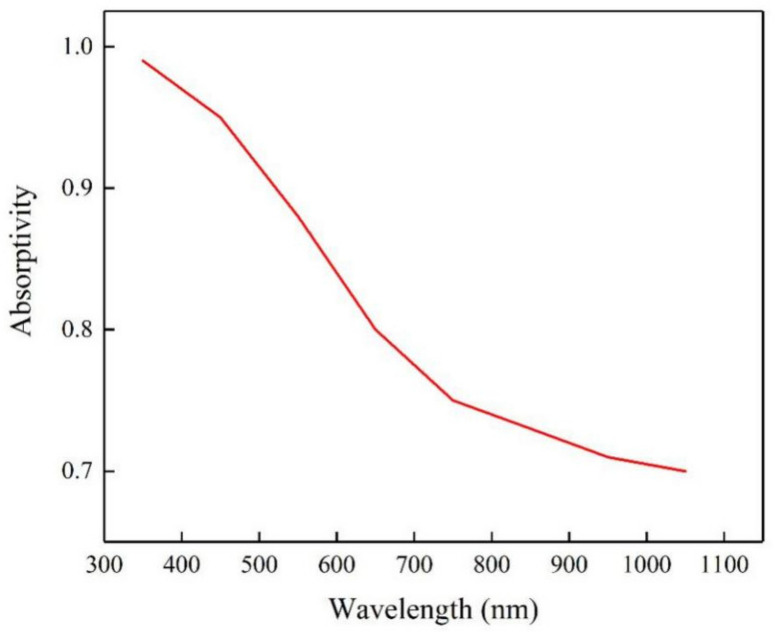
Absorption rate of diamond to the laser with different wavelength.

The absorptivity of the diamond material to the laser is affected by many factors. The ultraviolet spectrophotometer was adopted to measure the absorption rate of diamond in the laser beam of different wavelength bands. As shown in Figure 4, diamond material has a different laser absorptivity for different wavelength bands. From infrared wavelength to ultraviolet wavelength, the wavelength is shorter and the absorptivity of diamond for the laser is higher. Furtherly, different absorptivity can cause different processing mechanisms of a laser to the diamond. At the same time, the laser with different pulse width also has different effects on the removal of material. Therefore, it is critically important to comprehend the effects of diverse lasers on the diamond. Considering the types of lasers in the field of micromachining, this study mainly concerns the infrared band femtosecond laser (FSL) with a wavelength of 1064 nm, the green band picosecond laser (PSL) with a wavelength of 532 nm and the UV nanosecond laser (NSL) with a wavelength of 355 nm.

The main heat transfer is due to the heat conduction when the laser irradiates on the diamond. In the case of heat conduction, Fourier’s law of heat transfer can describe the temperature distribution of material in space and its change over time. The corresponding 2-D model was established with the solid heat transfer module in the commercial software of Comsol Multiphysics (5.4, Comsol Inc., Stockholm, Sweden). To simplify the model, some assumptions are indispensable. The material’s removal process mainly considers the thermal effect and ignores the chemical effect. In laser micromachining, plasma generation do not affect the heat absorption. The material is vaporizing and removed at once when the temperature of material reaches the vaporization point. Then, a Gaussian heat source model was established to simulate the laser source.

When laser irradiates on the material, the material cannot absorb the laser energy completely. Most of the incident laser will be absorbed, some will be reflected by the surface, some will be lost when laser pass through the material and some will be scattered by the surface. According to energy conservation, the relationship between material absorption and propagation of laser beam is:

*A* + *R* + *T* + *S* = 1 (1)

In this equation, *A* is the absorption rate of material to the laser, *R* is the reflectivity of material to the laser, *T* is light transmittance of material, *S* is the scattering coefficient of material.

For diamond, the *T* and *S* can be neglected. The Equation (1) can evolve to the following equation:

*A* + *R* ≈ 1 (2)

**Figure 5:**
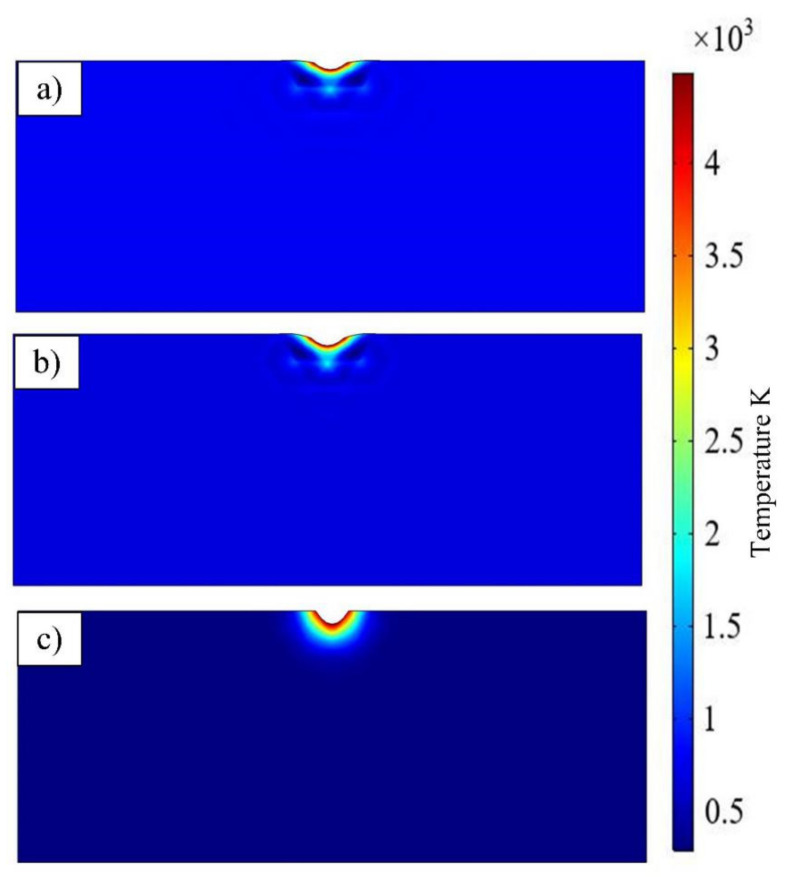
Comparison of the ablation and temperature distribution after one pulse of (**a**) FSL, (**b**) PSL and (**c**) NSL irradiation.

The Gaussian flux was applied on the surface of the model, and the surface emissivity was set. The different absorption coefficients were also set according to the absorption rate of different lasers by diamond. The parameters of different lasers are listed in Table 2. After calculation, the simulation results illustrate the material removal and the temperature field distribution as shown in Figure 5. The pit morphology and temperature field distribution after the three kinds of laser are nearly v-shaped, which determined by the temperature distribution of the Gaussian laser (the temperature decreases gradually from the center to the outside). It also found that the pit morphology of NSL is more positive and deeper than FSL and PSL.

**Figure 6:**
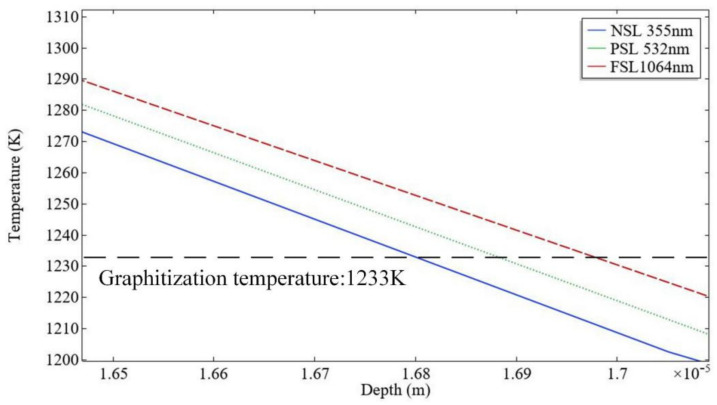
Comparison of graphitization depth under FSL, PSL and NSL irradiation.

The diamond will transform into graphite while the temperature reaches 1233 K and graphite will be removed by gasification at 4827 K [15]. Thus, it can consider that the material with a temperature between 1233 K and 4827 K is the graphite layer. A 2D intercept line was used to measure the depth from the pit to the bottom of the model. The depth can represent the phase transition degree. In Figure 6, the degree of graphitization of NSL is similar to the ultrafast laser, and the graphite layer is thinner than FSL and PSL. That is because the diamond has an extremely high absorption of laser energy with an ultraviolet band, which results in the photon energy being strong enough to break directly the molecular bonds of material to etch the material through photochemical mechanism instead of ablation which occurs when infrared band or green band laser radiate on diamond [16,17,18,19,20,21]. Thus, UV nanosecond laser has superior results. This simulation substantiates that it mainly depends on laser wavelength rather than the pulse width. The wavelength of the laser governs the processing mechanism and phase transition of the diamond. Furthermore, nanosecond has higher efficiency than femtosecond and picosecond laser. Therefore, UV nanosecond is more positive to fabricate the PCD skiving cutter.

The simulation is carried out in 2D symmetry model, which can lead to quantitative assessment of laser exposure induced graphitization. The simulate plate is selected on the symmetry plane of the laser load of the PCD workpiece.

### 3.2. Influence of Laser Processing Parameters on Contour Accuracy

**Figure 7:**
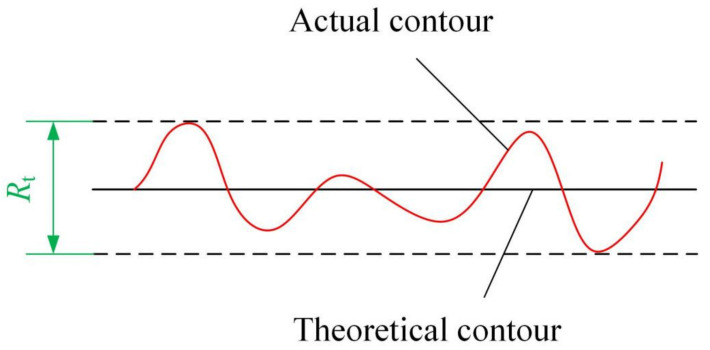
Schematic of *R*_t_ measurement.

The contour accuracy of the cutting tool has a crucial impact on the performance of the cutting tool, and it is one of the significant standards for evaluating the quality of laser cutting. Here, *R*_tp_ is defined as the contour accuracy of the cutting edge, which is numerically equal to the maximum peak-valley deviation between the actual contour and theoretical contour, shown in Figure 7. The laser scanning line interval, defocusing amount and scanning speed are the main parameters that affect the *R*_t_ as the laser power is constant. Hence, this section investigated the influence of laser scanning line interval, defocusing amount and scanning speed on the *R*_t_ according to the processing method shown in Figure 2, and detected the processing results through the scanning electron microscope.

**Figure 8:**
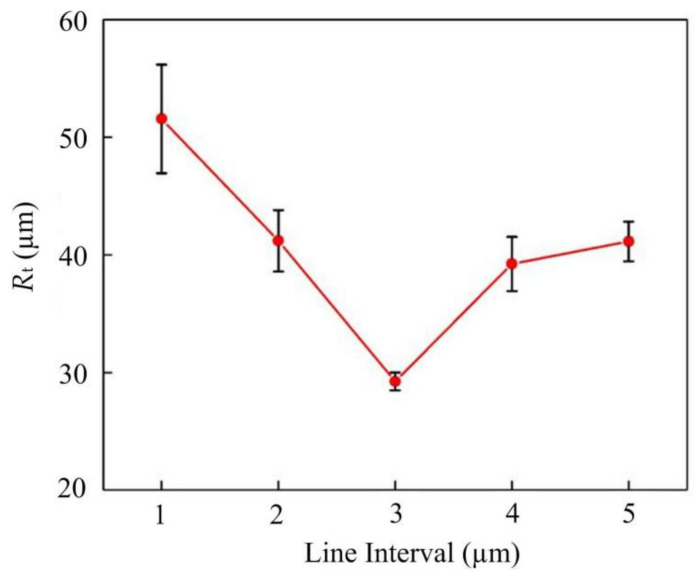
The effect of line interval on the *R*_t_.

**Figure 9:**
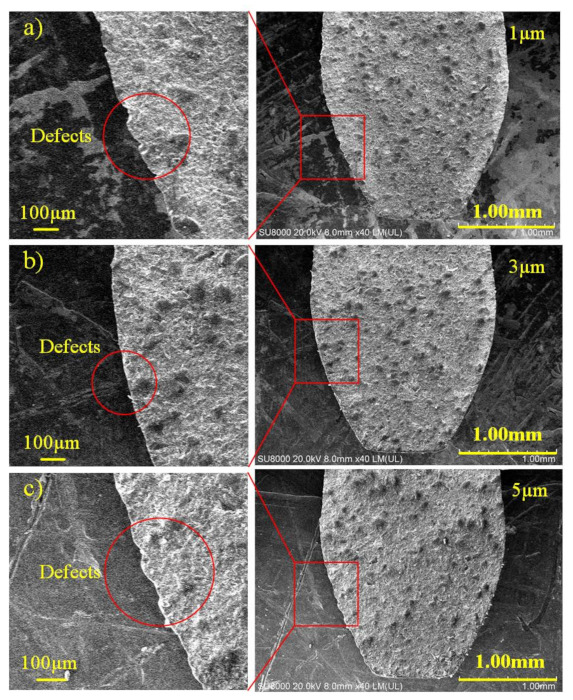
The SEM images of cutting edge of PCD skiving cutter at different line interval: (**a**) 1 µm, (**b**) 3 µm, (**c**) 5 µm.

The effect of laser scanning line interval on *R*_t_ at a laser power of 10 W, scanning speed of 500 mm/s and defocusing amount of 0, is shown in Figure 8. As the line interval increases, the *R*_t_ decreases gradually at first and then increases. The *R*_t_ has a minimum value when the line interval is 3 µm in Figure 9b, the defect is very small. However, when decreasing the line interval, the overlap rate of the laser spot increases. The energy density raises accordingly, resulting in excessive etching at the cutting edge. In Figure 9a, there are a few defects at the edge as the line interval is 1 µm. Similarly, the overlap rate is undersize when the line interval is too large. The energy density reduces accordingly, in result incomplete removal of the material at the cutting edge, which causes the raising of the *R*_t_ value further, such as the defects shown in Figure 9c.

**Figure 10:**
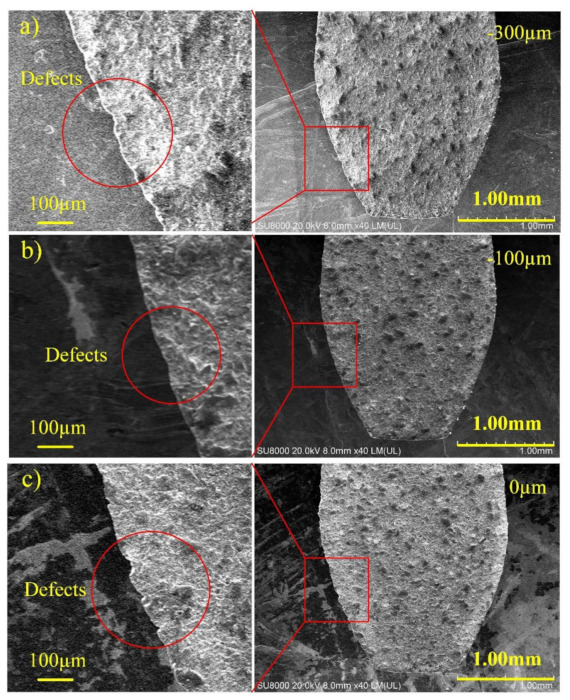
The SEM images of cutting edge of PCD skiving cutter at different defocusing amount: (**a**) −300 µm, (**b**) −100 µm, (**c**) 0 µm.

**Figure 11:**
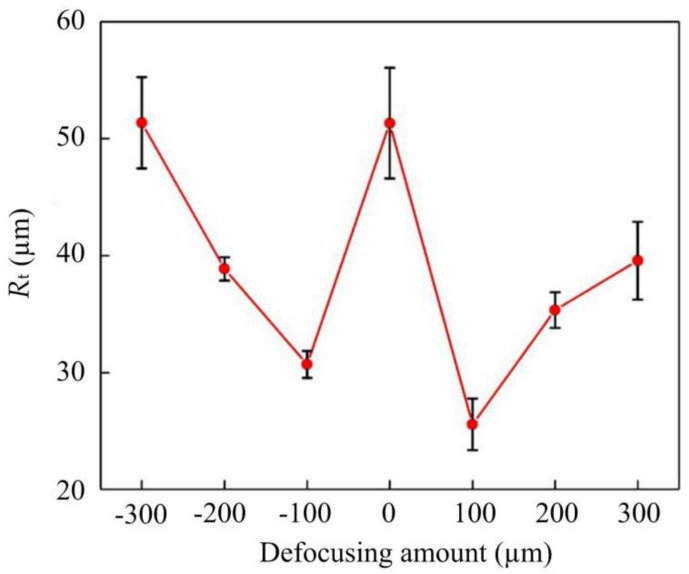
The effect of defocusing amount on the *R*_t_.

The SEM image of the cutting edge at a laser power of 10 W, a line interval of 1 µm, a scanning speed of 500 mm/s and a negative defocusing amount of −300 µm is demonstrated in Figure 10a. The defects are not negligible. The reason for the obvious defects is that the focus point is far from the cutting edge, the energy density irradiated on the edge is low so that the material cannot remove uniformly. When decreasing the defocusing amount, the average power increases with a better result. It can be seen in Figure 10b, the defects are reduced obviously. However, when further moving the focus point up, the accuracy becomes worse in Figure 10c. This change largely results from the fact that the energy density interacted on the edge heighten, which results in excessive etching. Further, the experiment was also applied under the positive defocus condition. The effect of the defocusing amount is exhibited in Figure 11. The result reveals an analogic influence. The *R*_t_ value decreases first and then increases, regardless of whether the defocusing amount is positive or negative. In addition, the *R*_t_ value is smallest at a positive defocusing amount of 100 µm. This result reveals an appropriate defocusing amount can reduce the laser energy density on the cutting edge and achieve a favorable processing result.

**Figure 12:**
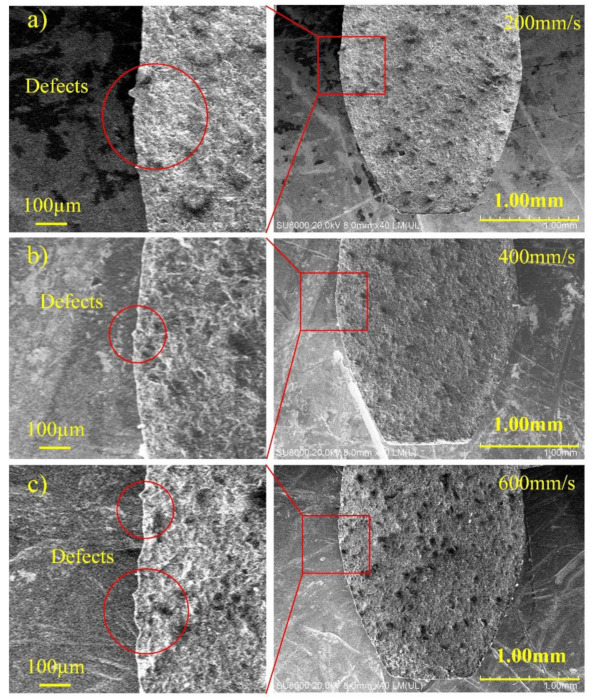
The SEM images of cutting edge of PCD skiving cutter at different scanning speed: (**a**) 200 mm/s, (**b**) 400 mm/s, (**c**) 600 mm/s.

**Figure 13:**
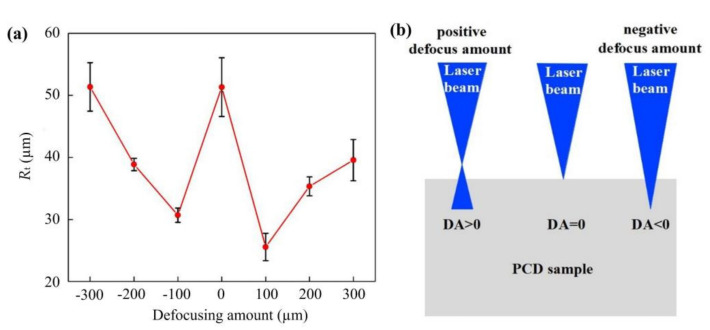
(**a**) The effect of scanning speed on the *R*_t_ and (**b**) the definition of defocus amount.

The SEM images of the cutting edge under different scanning speed are shown in Figure 12. The effect of scanning speed on *R*_t_ at a laser power of 10 W, a line interval of 1 µm and a defocusing amount of 0, is manifested in Figure 13. The results indicate that *R*_t_ gradually decreases first and then increases with the increase of scanning speed. The reason is analogic that the spot overlap rate raises with the decrease of scanning speed. It causes a great energy density at the cutting edge, resulting in excessive etching and poor accuracy. However, the energy at the cutting edge is insufficient when the scanning speed is too high, so the *R*_t_ increases accordingly.

The above-mentioned research presents that the processing parameters govern the contour accuracy of the cutting edge. A positive cutting edge can be obtained by adjusting different processing parameters to regulate the energy density for acting on the cutting edge.

### 3.3. Finishing Processing

**Figure 14:**
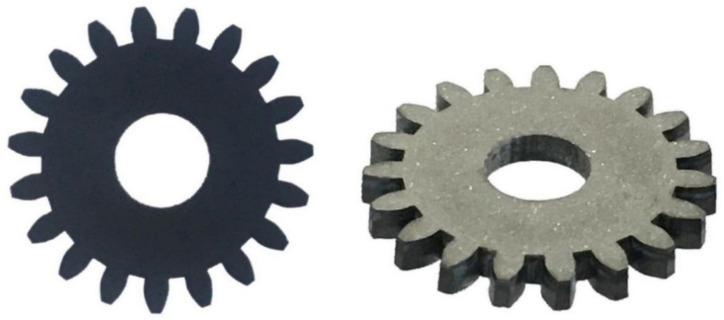
The material object images of PCD skiving cutter.

**Figure 15:**
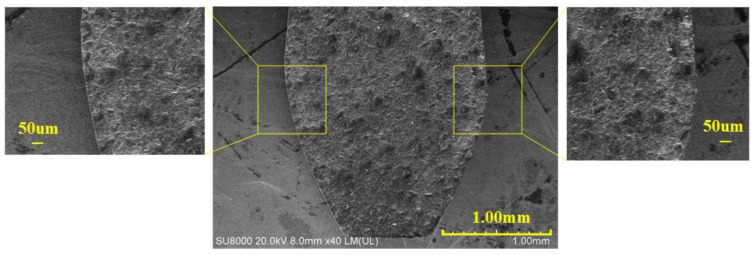
The SEM image of cutting edge of PCD skiving cutter.

We can use the above-mentioned parameter combination to make the PCD blank into rough shape. Furtherly, finishing the tool by the finishing machining method described in Figure 3. The PCD skiving cutter processed is shown in Figure 14. The single tooth cutting edge is described in Figure 15. It indicates that the cutting edge is flat without any cracking defects. Moreover, the *R*_t_ value reaches 5.6 µm after finishing processing.

### 3.4. Raman Spectroscopy Analysis

**Figure 16:**
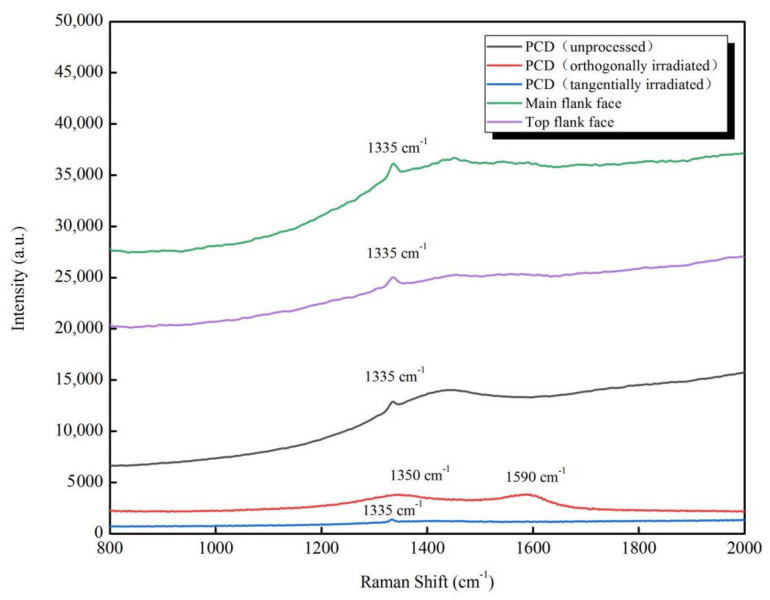
Raman spectroscopy on different conditions.

The Raman spectroscopy was used to investigate the effect of different cutting methods on the phase transition of the diamond and characterized the flank face of the PCD skiving cutter to extrapolate the influence of laser cutting on cutting tool. Figure 16 gives the Raman spectroscopy in a few conditions. For unprocessed PCD blank (black curve), there is only a peak at 1335 cm^−1^, which represents the sp^3^ (diamond-like) hybrid structure. After orthogonally irradiation (red curve), there exists a peak in D-band at 1350 cm^−1^ and G-band at 1590 cm^−1^. It indicates a mixture of carbon phase with disorder and sp^2^ (graphite-like) hybrid structure. In contrast, the spectrum of tangentially irradiated (blue curve) only has the diamond phase. The reason for this difference is that the laser is in tangential contact with the material for the tangentially irradiated, the energy density absorbed by the material is limited. It is similar to the laser polishing, and the subsequent UV laser can remove the graphite component caused by the previous laser. This research provides evidence for the practicability of the skiving cutter processing technology.

For further proving this research, a Raman test of the main flank face and top flank face generated by a two-step machining demonstrated as before is significant. As expected, the Raman spectroscopy (green and purple curve) exhibits only an obvious peak at 1335 cm^−1^ for both two faces, indicating a strong sp^3^ bonds, respectively. Therefore, the two-step fabrication process is feasible to obtain an PCD skiving cutter without phase transition.

## 4. Conclusions

In this study, a two-step laser machining process combining roughing machining and finishing machining was proposed and performed with UV nanosecond laser. During the roughing machining, the orthogonally irradiation was adopted to make a rough shape of the skiving cutter. Subsequently, tangentially irradiation was used in finishing machining to process a high-accuracy cutting edge. The simulation results indicate UV nanosecond laser can acquire a more positive cutting result with less graphitizing, flatter morphology. In experiments, *R*_t_ value decreases first and then increases as the increase of the line interval, scanning speed and defocusing amount (no matter positive or negative defocus). A minimum of *R*_t_ can be received at a line interval of 3 µm, a positive defocusing amount of 100 µm and a scanning speed of 400 mm/s. Through the Raman spectrum of the flank face of the PCD skiving cutter, the cutting face on a diamond has no graphite phase transition with the UV nanosecond laser machining. In addition, after the two-step machining, a superior PCD skiving cutter with an *R*_t_ of 5.6 µm was obtained in this study.

## Figures and Tables

**Table 1:** Parameters of UV nanosecond laser.

Input Laser Parameters	Values
Pulse Width	10 ns
Wavelength	355 nm
Pulse Repetition Rate	40–150 kHz
Focal Spot Diameter	20 µm
Average Power	<12 W
Focus Point Diameter	20 µm
Divergence Angle	1.2 mrad

**Table 2:** Parameters of the FSL, PSL, NSL used in the simulation.

	FSL	PSL	NSL
Pulse Width	120 fs	10 ps	10 ns
Wavelength	1064 nm	532 nm	355 nm
Pulse Repetition Rate	200 kHz	1 MHz	50 kHz
Pulse Energy	100 µJ	100 µJ	200 µJ

## Data Availability

The data presented in this study are available on request from the corresponding author.
